# Clinical Cases in the Medical Wards of Mercy Hospital, June 5, 1866

**Published:** 1866-07

**Authors:** N. S. Davis

**Affiliations:** Prof. Practical and Clinical Medicine, Chicago Medical College


### The Clinique.

### CLINICAL CASES IN THE MEDICAL WARDS OF MERCY HOSPITAL, JUNE 5TH, 1866.

BY N. S. DAVIS, M.D., Prof. Practical and Clinical Medicine, Chicago Medical College.

CASE I. *Chronic Ague; Enlargement of Spleen; Cough.*—This patient is a boy, aged about 16 years, who was admitted to the Hospital about three weeks since. Those members of the hospital class who were in attendance at that time will remember examining him carefully, soon after his admission. His skin was bloodless; lips, tongue, and gums pale, indicating a decidedly spanæmic or impoverished condition of the blood, especially in reference to the red corpuscles; pulse soft, and nearly natural in frequency; appetite impaired; and he complained of a harsh cough; with some soreness in the chest; and great muscular debility. He had suffered paroxysms of intermittent fever, at irregular intervals, for several weeks. You will also remember, that a physical exploration by auscultation, percussion, and palpation, revealed a mixture of dry and moist rhonchi in both sides of the chest, and extensive dulness over the left hypochondriac region, extending from the seventh rib to near the anterior part of the crest of the ilium; the first, indicating chronic bronchitis, and the latter, a decided enlargement of the spleen. The pathological conditions then presented by the patient were, impaired tonicity of the organized structures with that impoverishment of the blood which usually results from chronic ague, complicated with a low grade of inflammation in the bronchial mucous membrane, and enlargement and induration of the spleen. To meet the indications for treatment afforded by the general impairment of tonicity, and irregular paroxysms of ague, the patient was directed to take three grains of sulphate of quinine after breakfast and dinner each day. For the bronchial irritation and enlargement of the spleen, he was directed to take a teaspoonful of the following mixture before each mealtime and at bedtime:—

℞. Muriate of Ammonia,------------------ ʒiij.
Tart. Ant. et Pot.,---------------------- gr.ij.
Sulph. Morph., ------------------------ gr.iij.
Syrup of Liquorice,--------------------- ℥iv.

Mix.

The muriate of ammonia in the mixture was represented as a valuable alterative, well calculated to promote absorption of the adventitious deposits or exudations into the spleen, without any of the objectionable properties possessed by the preparations of mercury; while the small doses of antimony and morphine would lessen both the irritability and vascularity of the bronchial mucous membrane. He followed the foregoing treatment for one week, with a constant improvement in all his symptoms. The quinine was then limited to one dose after breakfast each morning, and the muriate of ammonia mixture continued four times a-day as before. At the end of the second week, his cough was entirely removed, and his strength, color, and appetite so much improved that he ceased to present himself for further advice until to-day.

You see, now, his general aspect very much changed. His prolabia are red; the veins of the surface moderately full; and his flesh increased. He has had no cough, appetite good, and bowels regular; but during the last few days he has complained of several attacks of pain in the epigastrium, apparently of a neuralgic character. We say neuralgic, because they were accompanied neither by fever, loss of appetite, or flatulency. By re-examining the epigastric and left hypochondriac regions at this time, you find nothing unnatural in the epigastric region proper, but dulness still exists over too large a portion of the hypochondrium. And by placing the patient in the dorsal position, with thighs flexed upon the pelvis so as to relax the abdominal muscles, the fingers pushed a little deeply under the margin of the ribs on the left side readily feels the rounded hard margin of the spleen; thus showing that it is still larger and harder than natural. The enlargement is very much less than when admitted into the hospital, three weeks since, but still sufficient to impair the functions of that organ, and, indirectly, that of the stomach also. It is quite probable that the paroxysms of epigastric pain, of which he complains, have originated from taking food more freely than the digestive organs could digest perfectly. The leading indications for treatment at present are, to improve the functions of the stomach and still further reduce the enlargement of the spleen. To fulfil these indications, we will give the patient one of the following pills before each mealtime and at bedtime:—

| | | |
|---|---|---|
| ℞. | Ext. Hyoscyamus, | 40 grs. |
| | Ext. Taraxacum, | 40 grs. |
| | Sulph. Ferri, | 20 grs. |
| | Pillulæ Hydrarg., | 20 grs. |

Mix, and divide into 40 pills.

The hyoscyamus and iron are designed to improve the sensibility and secretory action of the stomach, while the taraxacum and blue mass will continue such alterative action as will further reduce the enlargement of the spleen. The food of the patient should be plain, easily digested, and taken in very moderate quantities at a time.

CASE II. Mr. B., aged 35 years, a native of Ireland, was admitted into the hospital eight days since. He is addicted to the excessive use of alcoholic stimulants, and was exhibiting the initial symptoms of delirium tremens at the time of admission into the hospital. His mind was despondent and apprehensive; frequent startings; insomnia; and loss of appetite.

To control these symptoms, he took the tincture of Cannabis Indica, in doses of 20 drops, every four hours, and small quantities of beef-tea at suitable intervals. Under this treatment the nervous symptoms so far subsided that the patient slept well, recovered a moderate appetite, and lost all that starting and apprehensive state of mind characteristic of ordinary delirium tremens. Still, a week has elapsed since the amount of improvement just stated, and the patient instead of being up ready for a discharge, as is usual in most cases of this kind, is here in bed, presenting a train of obscure symptoms which require the most careful attention of the physician. The mind presents a degree of torpor or vacuity, and a forgetfulness that often causes him to give erroneous answers to questions, and sometimes to exhibit slight wandering for a few minutes at a time. The skin is cool and relaxed, but the pulse constantly up to 100 per minute; the tongue moist, pointed, and tremulous; little or no appetite; and though complaining of no pain in the abdomen, yet pressure on the epigastrium causes a frown that indicates plainly some tenderness. The fæcal and urinary secretions are nearly natural.

The symptoms now presented by the patient indicate the existence of a grade of irritation in the gastric mucous membrane, such as causes passive congestion of the capillaries and suspension of the secretion of gastric juice, and, therefore, loss of appetite. The brain, which had suffered derangement both in its nutrition and sensibility during the period of excessive use of alcoholic stimulants, cannot readily regain its natural condition and steadiness of function, while the stomach fails to replenish the blood with the products of healthy digestion. You thus see not only a clear explanation of the symptoms at present existing, but also the further dangers to which the patient is exposed. The continuance of the impaired condition of the brain makes the patient constantly liable to, at least, temporary mental hallucinations, under the influence of which he might do injury to himself or others, and thereby make himself subject to accusations of crime by the majority of those around him. On the other hand, a continuance of the morbid condition of the gastric mucous membrane would lead either to softening of its texture, or chronic follicular inflammation. To obviate these tendencies and restore the patient to health, we must adopt such treatment as will allay the morbid condition of the stomach, and thereby renew the processes of digestion and assimilation. This will result in furnishing the brain with the restorative influence of nutritious and healthy blood, and its functions will soon become steady and normal. There are several formulæ well adapted to accomplish this result, so far as medicine is needed. The following are such as I have frequently used with benefit:—

℞. Nitras Argenti,---------------------- 10 grs.
Ext. Hyoscyamus,------------------- 30 grs.

Mix, and divide into 30 pills, one of which should be given before each mealtime and at bedtime.

℞. Oxide of Zinc,---------------------- 30 grs.
Lupulin,---------------------------- 20 grs.

Mix, and divide into 12 powders, one of which may be given before breakfast, dinner, and at bedtime.

℞. Sub. Nit. Bismuth,------------------- ʒj.

Lupulin,---------------------------- 18 grs.

Mix, and divide into 12 powders, one of which may be taken before each meal, and at bedtime.

For the patient before us, we shall direct the pills mentioned in the first of these prescriptions, to be given, one each morning, noon, teatime, and bedtime. He should be induced, also, to take a moderate amount of plain nourishment, such as milk, rice, beef-tea, etc., at each mealtime.

The results of this treatment, you will learn at a future clinic hour.

NOTE.—After using the remedies and diet indicated above eight or ten days, the patient had recovered a moderate appetite, a cheerful expression of mind and countenance, but his muscular strength improved very slowly. The nitrate of silver was then discontinued, and strychnine, in doses of one-twentieth of a grain substituted in its place, under which his recovery was slowly completed.

